# Effectiveness of Elastodontic Devices for Correcting Sagittal Malocclusions in Mixed Dentition Patients: A Scoping Review

**DOI:** 10.3390/dj12080247

**Published:** 2024-08-02

**Authors:** Rebecca Ureni, Alessio Verdecchia, Carlota Suárez-Fernández, Manuela Mereu, Roberto Schirru, Enrico Spinas

**Affiliations:** 1 Department of Surgical Sciences, Postgraduate School in Orthodontics, University of Cagliari, 09124 Cagliari, Italy; rebeure@yahoo.it (R.U.); manumereu@yahoo.it (M.M.); bobschirru@gmail.com (R.S.); 2 Department of Surgery and Medical-Surgical Specialities, School of Medicine and Health Sciences, University of Oviedo, 33003 Oviedo, Spain; suarezcarlota@uniovi.es

**Keywords:** interceptive orthodontics, malocclusion, myofunctional therapy, elastodontic devices, mixed dentition

## Abstract

Elastodontics is an interceptive orthodontic therapy that uses light and biological elastic forces through preformed or custom-made removable orthodontic appliances. This study aims to evaluate the effects of elastodontic devices on correcting sagittal discrepancies in growing subjects with mixed dentition. Electronic research was conducted on four databases: PubMed, Scopus, EMBASE, and Web of Science. Data were extracted based on the first author, year of publication, setting and country, study design, sample characteristics, sample size calculation, type of malocclusion, intervention, control group type, compliance, follow-up, and cephalometric measurements. Sixteen studies were included in the final review. Most studies observed a statistically significant reduction (*p* < 0.05) in SNB and ANB angles. Ten studies reported a reduction in overjet, while eight studies found no change in facial divergence. Comparisons with conventional functional devices revealed no consensus on the skeletal and dentoalveolar effects. Elastodontic appliances significantly improve cephalometric and dentoalveolar parameters, potentially correcting skeletal and dental relationships. However, result variability and unclear advantages over traditional appliances highlight the need for further research.

## 1. Introduction

Early intervention for malocclusion during the mixed dentition phase is highly recommended, particularly in cases where alterations in sagittal relationships present a significant risk of dental trauma to the upper incisors [1]. Interceptive orthodontic therapy represents a preventive approach to treating malocclusion in pediatric patients. It is based on the understanding that signs of various malocclusions are frequently identifiable during both early and late mixed dentition stages [2,3] and that they do not self-correct with age [4]. However, a consensus on the effectiveness of interceptive therapy has not been reached. Some studies suggest that early treatment may lead to stable occlusion, while others indicate that children would not benefit from early treatment aside from an increase in self-esteem [5,6,7].

Interceptive treatment becomes particularly relevant when addressing factors that disrupt the harmonious development of the maxillary and mandibular arches, often leading to skeletal and dento-alveolar compensations to maintain stable function and occlusion. Elastodontics is an interceptive orthodontic therapy that utilizes light and biological elastic forces through preformed or custom-made removable orthodontic appliances crafted from biomedical silicone or other elastic materials. These devices are activated by the patient’s muscle function to correct malocclusions in growing patients, aiming to eliminate functional disturbances, correct tooth positions, and potentially influence growth [8,9].

The material of elastodontic devices facilitates orthodontic movement in synergy with the neuromyofascial system, while the vestibular flanges prevent perioral muscles from affecting tooth movement. Previous evidence suggests that elastomers could be effective in promoting significant clinical improvement in early signs of malocclusions such as crowding, overbite, overjet, and sagittal molar relationships. These devices are primarily designed for the treatment of orthopedic–orthodontic issues during the developmental age and, therefore, are used in deciduous or mixed dentition [10].

Nowadays, orthodontists have access to a wide range of easy-to-wear devices that act comprehensively on the stomatognathic system, seamlessly integrating with the neuromuscular system and requiring fewer patient check-ups [11]. These devices exert three-dimensional effects on all structures of the stomatognathic apparatus, correcting functional issues of soft tissues and promoting the restoration of oral, perioral, and lingual muscle function [12,13].

No previous scoping reviews have evaluated the outcomes of elastodontic devices using cephalometric measurements. Therefore, this study aims to assess the effects of elastodontic devices on correcting sagittal discrepancies in growing subjects with mixed dentition through cephalometric evaluation. A secondary objective is to compare the outcomes of these devices with conventional orthodontic devices and untreated groups.

## 2. Materials and Methods

The review was conducted in adherence to the protocol established by the Preferred Reporting Items for Systematic Reviews and Meta-Analyses extension for conducting Scoping Reviews (PRISMA-ScR) [14]. This scoping review was not registered.

To define the parameters of the research strategy, we formulated a primary research question, “What is the dento-skeletal effect of elastodontic devices on the saggittal plane in mixed dentition patients?”

The search was conducted on 13 May 2024, using the Scopus, Web of Science, Embase, and PubMed databases. The search strategy is reflected in Table 1.

The studies were included if they met the following criteria, reported according to the PICO format: studies involving growing human subjects in the mixed dentition period (intervention); studies evaluating the treatment effects of elastodontic devices (intervention); studies comparing before and after treatment outcomes with elastodontic devices against other functional appliances or untreated patients (comparison); and studies assessing dental and/or skeletal outcomes using teleradiographic data (outcomes).

The inclusion criteria were as follows:

- Patients in mixed dentition
- Randomized controlled trials
- Retrospective and prospective studies

The exclusion criteria were:

- Patients with permanent or full deciduous dentition
- Studies without radiographic records
- Review studies

There were no limitations to the publication year or language.

Duplicated records were removed using Zotero and then verified manually. Subsequently, two reviewers (AV and CSF) independently evaluated and selected valid studies based on titles and abstracts.

After the full-text assessment, the studies to be included in the review were selected. In case of disagreement, a third reviewer (RU) resolved the issue.

For each study, we collected the following information: first author, year of publication, setting and country, study design, sample characteristics (sex, age), sample size calculation (yes/no), type of malocclusion, intervention (device and wear instructions), control group type, compliance, follow-up, and cephalometric measurements.

## 3. Results

**Figure 1:**
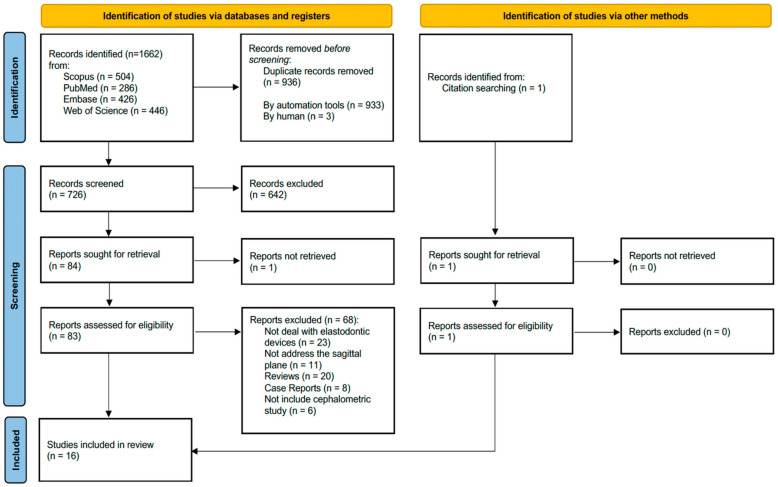
Flowchart of study selection process [15].

The initial search yielded a total of 1662 results: 504 from Scopus, 286 from PubMed, 426 from Embase, and 446 from Web of Science. After removing duplicates, 726 articles remained. Subsequently, after reading the title and abstract, 83 articles underwent full-text evaluation. Finally, after a thorough assessment, 16 articles met the inclusion criteria and were included in the review. The process of literature search and selection is illustrated in a flow diagram presented in Figure 1.

### 3.1. Study Characteristics

The study characteristics, such as author, year, setting, country, study design, and conclusions, are outlined in Table 2.

Regarding the country, Italy has the highest number of studies on the topic (*n* = 8) [12,16,17,18,19,20,21,22], followed by China (*n* = 3) [23,24,25] and Turkey (*n* = 3) [26,27,28]. One study is from India [29], and another is from Egypt [30].

Most of the studies included in this review are recent, being published in 2021 (*n* = 6) [12,16,17,25,26,29], 2022 (*n* = 5) [18,19,20,23,24], and 2023 [21,27,30]. Only one study was published in 2004 [28].

According to the study design, 12 studies were retrospective [12,16,17,18,19,21,22,23,24,25,27,28], while 4 were prospective [20,26,29,30]. Most of these studies (*n* = 14) included a control group for comparison [12,16,17,19,20,21,22,23,24,25,27,28,29,30].

**Table 2:** Characteristics of included studies.

Author (Year) [Reference]	Setting/Country	Study Design	Conclusions
Chen LR et al. (2022) [23]	Taichung Veterans General Hospital/Taiwan	Retrospective Case–Control Study	EF group: UI angle decrease, LI angle increase, and LI tip to Mb plane distance decrease in comparison to the control group UI angle changes were statistically significant One-year follow-up was not sufficient to determine the skeletal effect of the EF appliance
Ciavarella D et al. (2021) [16]	Orthodontic Department, University of Foggia/Italy	Retrospective Case–Control Study	EA treatment produces the following: No significant dental or aesthetic changes Minor skeletal effects Mb length and LFH increased after EA treatment compared with untreated patients
Ciftci V et al. (2021) [26]	Department of Orthodontics, Faculty of Dentistry, Cukurova University/Turkey	Prospective Study	Multi-P functional appliance: Reduction in OVJ, OVB, and convexity in the mixed dentition stage Follow-up data are needed to evaluate the long-term benefits of this appliance
Çoban Büyükbayraktar Z et al. (2023) [27]	Orthodontic Department of Sivas Cumhuriyet University Dentistry Faculty/Turkey	Retrospective Case–Control Study	TB and myobrace can be used for Mb advancement Twin-block appliance was more effective The long-term effects of myobrace on Mb advancement are unknown
Fichera G et al. (2021) [12]	Department of Orthodontics, University of Catania/Italy	Retrospective Case–Control Study	EA showed: Improvement of OVJ, OVB, crowding, and the sagittal molar relationship EA is a simple, natural, and less invasive therapeutic option for treating malocclusion
Galluccio G et al. (2021) [17]	Italy	Retrospective Case–Control Study	Occlus-o-Guide^®^, FR-2, TB increase in Mb length FR-2 and TB are more effective in increasing the Mb length The reduction in the ANB angle was similar in the three groups, but the increase in the SNB angle was significant only for FR-2 and TB Occlus-o-Guide^®^, FR-2, and TB produce the following: Reduction in OVJ and OVB in relation to the control group The reduction produced by TB was significant compared to that for the other two devices The IMPA angle increased more in the O-o-G^®^ group The esthetic analysis shows the following: TB group: More reduction in facial convexity More reduction in the thickness of LL O-o-G^®^ group: More retrusion of UL followed by that in the TB group FR-2 group: Increase in the thickness of the UL compared to the control group
Inchingolo AD et al. (2022) [18]	Italy	Retrospective Study	The AMCOP^®^ Integral with a flat mastication plane is sufficient to correct mild hyperdivergency The AMCOP^®^ Open is more indicated in severe hyperdivergent. This device also contributes to the functional re-education of the tongue The AMCOP^®^ SC allows the correction of class II dysmorphism favoring a mandibular advancement The long-term stability of the results obtained is still to be evaluated
Johnson JS et al. (2021) [29]	KVG Dental College and Hospital, Sullia, Karnataka/India	Randomized Control Prospective Study	TB and myobrace appliances: Not effective in restricting the forward growth of the maxilla TB produced the following: Significant skeletal and dentoalveolar changes Better improvement of mandibular growth (Go–Ar, Go–Me, Ar–Gn) than the myobrace system Myobrace induced the following: Reduction in OVJ, forward rotation of the mandible, and forward positioning of the mandible TB appliances demonstrated the following: Correction of full Class II molar relationship Better correction of molar relation than myobrace TB and myobrace showed the following: Improvement in the profile TB showed the following: Increase in the anterior and posterior facial heights Myobrace group showed the following: Better bite closure effect Myobrace and TB groups exhibited the following: Flaring of the lower incisors, such as unfavorable treatment outcome More prominent in the myobrace group than the twin-block group
Lanteri V et al. (2022B) [19]	Department of Biomedical, Surgical and Dental Sciences, University of Milan/Italy	Randomized Control Retrospective Study	Customized and preformed EGAs showed the following: Improvement of Class II malocclusion and anterior crowding Reduction in OVJ and OVB Significant changes regarding the sagittal and vertical cephalometric relationship Customized EGA was as follows: More effective in correcting anterior crowding, dento-skeletal vertical relationship, and the position of the permanent incisor compared to the preformed appliance
Lo Giudice A et al. (2022) [20]	Department of Orthodontics, Pediatric Unit, University of Catania/Italy	Prospective Control Study	EAs showed the following: Improvement in OVJ, OVB, crowding, and the sagittal molar relationship compared to controls EAs determined the following: Correction of early signs of malocclusion in Class II subjects Harmonious development of the palate
Madian AM et al. (2023) [30]	Orthodontic Department, Faculty of Dentistry, Alexandria University/Egypt	Randomized Control Prospective Study	TB was as follows: More effective than myobrace in improving the upper and middle airways No difference was detected regarding the lower airway TB and myobrace produced the following: Reduction in the severity of developing skeletal Class II due to mandibular retrognathism by forward posturing of the mandible
Patano A et al. (2023) [21]	Department of Orthodontics at the Policlinico of Bari/Italy	Retrospective Case–Control Study	AMCOP^®^ SC permitted the following: Correction of skeletal Class II malocclusion Improvement of Mb advancement Functional elastodontic device therapy determined the following: Significant airway changes in skeletal Class II subjects compared with an untreated control group Improvement of deglutition, phonation, and respiratory function The hyoid bone shifted inferiorly at the end of treatment in the treated group with respect to the control group
Ronsivalle V et al. (2023) [22]	Section of Orthodontics, School of Dentistry, University of Catania/Italy	Retrospective Case–Control Study	EAs permitted the following: Correction of Class III malocclusions in children Improvement of morphology of the palate in the transverse and anteroposterior directions Correction of anterior crossbite by promoting harmonious restoration of maxillary growth
Usumez S et al. (2004) [28]	Department of Orthodontics, School of Dentistry, Selcuk University, Konya/Turkey	Retrospective Case–Control Study	Preorthodontic trainer appliance caused the following: Better reduction in LI proinclination and OVJ than in the control group Increase in total facial height
Yang X et al. (2022) [24]	Pediatric Dentistry Department of Shanghai Ninth People’s Hospital/China	Retrospective Case–Control Study	Orofacial myofunctional therapy resulted in the following: Improvement of the patient’s lip strength A good option for mixed dentition patients with lip incompetence Preformed appliances determined the following: Improvement in lip strength and forward movement of the mandible LI protrusion had a negative effect
Zhang X et al. (2021) [25]	Department of Stomatology, The Second Affiliated Hospital of Jiaxing University, Jiaxing, Zhejiang Province/China	Retrospective Case–Control Study	Prefabricated myofunctional appliance and RME are as follows: The best option for the treatment of mouth breathers with Class II malocclusion in the mixed dentition period T4K showed the following: Optimal sagittal correction of maxilla and mandible Greater dental compensation with inhibition of skeletal remodeling Further studies with larger sample sizes are needed

AMCOP, Armonizzatori Multifunzionali Cranio-Occluso Posturali; ANB, PointA–Nasion–Point B angle; EA, elastodontic appliance; EF, Education Fonctionnelle; EAs, elastodontic appliances; EGA, eruption guide appliance; FR-2, Fränkel-2 appliance; LI, lower incisor; LFH, lower facial height; LL, lower lip; Mb, mandibular; OVJ, overjet; OVB, overbite; SNB, Sella–Nasion–Point B angle; TB, twin-block; UI, upper incisor; UL, upper lip.

The sample characteristics are detailed in Table 3.

The sample size was estimated in six of the studies [19,20,22,26,27,30]. Across the 16 studies, there were a total of 345 participants in the elastometric device group, comprising 142 males and 148 females; sex information was not reported for 55 patients. The mean age was provided in 15 studies, with a total mean age of 8.48 years. The control group comprised untreated patients in six publications [12,16,20,21,23,28], patients treated with another type of appliance in six studies [19,22,24,25,27,30], and both untreated and treated patients in two trials [17,29]. Ciftci et al. and Ichingolo et al. did not include a control group [18,26].

Patients included in 15 studies were Class II [12,16,17,18,19,20,21,23,24,25,26,27,28,29,30]. Among these, six studies focused on Class II Division 1 [17,23,25,26,28,29], and two studies also included Class I patients [19,24]. Ronsivalle V et al. studied Class III patients [22]. 

The follow-up period ranged from 6 months [27] to approximately 3 years [26].

#### Cephalometric Outcomes

Elastodontic Appliances

Intragroup differences and intergroup differences in the cephalometric values analyzed are detailed in Table 4.

The statistically significant skeletal effects of elastodontic therapy were evidenced by improvements in sagittal relationships. These improvements were indicated by an increase in the SNB angle in Class II subjects across 13 studies [12,17,18,19,20,21,23,24,25,27,28,29,30] and a reduction in the same angle in Class III subjects [22]. One study [16] did not analyze this parameter, and another study [26] reported non-significant results.

A statistically significant reduction in maxillary protrusion, indicated by the SNA angle, was observed in only two studies [21,25].

Most studies also reported significant improvements in skeletal class, demonstrated by a reduction in the ANB angle in 12 studies [12,17,18,19,20,21,23,24,26,28,29,30]; a reduction in a decrease in the ANB angle was noted in only one study [22]. In two studies, the effects on the ANB angle were not statistically significant [25,27].

The mandibular length was assessed using various cephalometric tracings and reference points. Despite low heterogeneity among studies, an increase in mandibular length was documented in eight papers [16,17,21,23,25,26,28,29]. This parameter was not evaluated in seven articles [12,18,19,20,22,24,30], and only one study [27] reported non-significant results.

The dentoalveolar effects most frequently analyzed were overjet (OVJ) and overbite (OVB). A statistically significant reduction in OVJ was observed in 10 studies [12,17,18,19,20,21,26,27,28,29], and a significant reduction in OVB was noted in four studies [12,17,19,20]. However, one study [22] reported a significant increase in OVJ in Class III subjects.

The direction of mandibular growth relative to the cranial base was altered, as indicated by a reduction in the SN-Go-Gn angle divergence in three studies [18,19,29]. In seven studies, this change was not statistically significant [16,17,21,23,26,27,28].

Analysis of the FMA angle showed that facial divergence remained largely unchanged, with no significant improvements reported [21,23,24,26,27,30]. This parameter was not mentioned in 10 studies [12,16,17,18,19,20,22,25,28,29].

#### Elastodontic Appliances vs. Other Functional Appliances

Neither Coban et al. [27] nor Madian et al. [30] found significant differences between the use of myobrace and twin-block appliances. In contrast, Johnson et al. reported statistically significant improvements with twin-block appliances in parameters such as Ar-Gn, OVB, and SN-Go-Gn compared to myobrace appliances [29].

Additionally, Galuccio et al. [17] observed a greater reduction in OVJ and OVB in patients treated with twin-block appliances compared to those using PMA Occlus-o-Guides.

Furthermore, Yang et al. noted a greater increase in SNB in patients treated with myofunctional preformed appliances compared to conventional early orthodontic appliances [24]. Similarly, Zhang et al. found that T4K showed better results than the Hyrax appliance, particularly in SNA, SNB, and Go-Me [25].

#### Elastodontic Appliances vs. Untreated Patients (Control)

In the study by Ciavarella et al. [16], a significant increase in mandibular size (Co-Gn) was observed in the PMA AMCOP group compared to the control group (*p* = 0.0173). However, the study by Patano et al. [21] found no significant changes in Co-Me (*p* = 0.102) using the same device. Galuccio et al. [17] observed a significant increase in the Ar-Pg distance when comparing the use of PMA Occlus-o-Guide to the control group (*p* = 0.004). Additionally, Johnson et al. reported significant changes in both Go-Me (*p* = 0.005) and Ar-Gn (*p* < 0.001) with myobrace use [29].

Only Galuccio’s study [17] revealed a significant difference in the SNA angle when comparing the use of PMA Occlus-o-Guide with controls (*p* = 0.017), indicating a reduction in this angle in treated patients. Similarly, Johnson’s study [29] found significant differences in SNB when comparing the myobrace with controls (*p* = 0.017). A smaller ANB was observed in the treatment groups of Patano [21] and Galuccio [17], suggesting mandibular advancement.

Regarding dental values, the reduction in OVJ was significantly greater in several studies [17,21,28,29], as was the reduction in overbite [17,21], when using elastomeric devices compared to the control.

## 4. Discussion

The literature resulting from this scoping review indicates that elastodontic devices, when used in growing patients with mixed dentition and mild-to-moderate sagittal issues, can facilitate partial or complete resolution of Class II malocclusions. These devices feature vestibular and lingual flanges with a central area for the teeth, which may have indentations, act as a positioner, or remain free to avoid constriction and orthodontic movement. The vestibular flanges function as lip bumpers and stimulate the bone proprioceptively, activating both arches in the vertical, transverse, sagittal, and torsional planes. The upper and lower planes can be positioned to promote mandibular advancement, while the occlusal plane can be adjusted to manage vertical dimension and tooth eruption. For atypical swallowing, a ramp and button on the lingual flange guide the tongue to the palate, aiding functional rehabilitation [8]. Similar to other functional orthodontic therapies, elastodontic devices induce a series of changes by stimulating muscle activity, which subsequently leads to skeletal and occlusal modifications [31]. This is supported by the results of the articles included in this scoping review, where a statistically significant increase in the SNB angle was observed in 13 out of 15 studies on Class II patients [12,17,18,19,20,21,23,24,25,26,27,28,29,30], with the SNB angle varying from +0.61° [27] to +2.82° [30]. Furthermore, a statistically significant reduction in OVJ was observed in 10 studies [12,17,18,19,20,21,26,27,28,29], ranging from −2.1 mm [21] to −4.58 mm [26]. Additionally, a significant decrease in the ANB angle, indicating mandibular advancement or at least a forward repositioning of the mandible, was found in 11 articles [12,17,18,19,20,21,23,26,28,29,30], with values ranging from −0.38 [27] to −2.42 [30]. These changes impacted not only the positional appearance of the mandibular–jaw and dentoalveolar complex but also the size of the mandible itself, with a noticeable longitudinal increase observed [16,17,21,23,25,26,28,29].

Although limited, an impact on the correction of facial divergence was also noted, yielding discrepant results. Some studies reported a reduction in vertical dimensions [18,19], while others observed an increase [29]. In most of the included studies, this parameter remained unaffected or exhibited statistically insignificant changes [16,17,21,23,24,26,27,28,30]. These findings reflect a substantial divergence of opinions in the literature. Some authors suggest that elastodontic appliances maintain unchanged lower facial height and facial proportions [32], whereas others report an increase in these measurements [33].

Only one study that evaluated the effect of elastodontic devices in Class III patients was retrieved [22]. Rosinvalle et al. found an increase in SNA, ANB, and OVJ and a decrease in SNB. These changes suggest that these devices can be used to successfully resolve anterior crossbite; however, the existing literature on this topic is insufficient. More studies are needed to clarify their use in these cases [22].

Comparing the results of our study with those of other articles reveals intriguing insights into the effectiveness of elastodontic devices relative to more traditional functional appliances. Multiple systematic reviews corroborate our study’s findings, indicating that elastodontic appliances (EAs) are more effective than no treatment in reducing overjet (OVJ), overbite (OVB), and mandibular crowding, as well as in establishing a Class I canine relationship. However, when compared to conventional functional appliances, EAs demonstrate lower efficacy in eliciting dental, skeletal, and soft tissue changes despite their cost-effectiveness [33,34,35].

A plausible explanation for the difference in mandibular skeletal effects between the two families of functional devices may be attributed to the material consistency. The high elasticity of elastodontic appliances (EAs) might make it challenging for young patients to maintain a protrusive mandibular position with the incisors in an edge-to-edge relationship [36].

The dentoalveolar and skeletal outcomes of EAs are contingent upon patient compliance. Adherence to instructions for wearing a removable appliance has been directly correlated with the treatment outcomes achieved [37]. Although the majority of studies recommend a protocol of 1 to 4 h of daily wear plus all night [12,16,18,19,20,21,22,23,25,26,29,30], few studies provide detailed information on whether the patients in their samples adhered to this therapeutic regimen [12,16,17,18,19,20,21,22,23,24,27,28]. Consequently, more rigorous studies are needed to monitor appliance usage accurately to determine patient compliance levels. Ultimately, EAs are designed to provide a combined effect, including guidance of tooth development, training of muscle function, and comprehensive early intervention [23]. They have proven effective in treating Class II mixed dentition patients with deleterious oral habits, such as atypical swallowing and altered lip strength [38]. The literature suggests that the most suitable period for this type of treatment is during the mixed dentition phase. Therefore, these devices can be effectively used for interceptive orthodontics in growing patients, particularly when the patient’s functional patterns are not optimal for harmonious maxillary base growth.

It is important to acknowledge that elastodontic devices cannot replace established orthopedic orthodontic treatments that have been validated by the literature. However, in cases involving growing patients with altered functions, elastodontic devices can serve as a valid alternative and an additional tool for orthodontists. These devices can support and guide growth by refunctionalizing the patient, thus achieving stable results. Therefore, case selection is crucial for the successful application of these devices.

While this scoping review includes a substantial number of studies, it has limitations that necessitate cautious interpretation of the results. Firstly, an analysis of the methodological quality of the included studies was not conducted. Second, the chemical and physical properties of the materials used were not detailed, and no structural differentiation of the various types of elastodontic appliances was made. Thirdly, the current literature is insufficient to deduce the effect of these devices in Class III patients. Fourth, the retrospective nature of the included studies is a limitation stemming from the inherent challenges in designing prospective studies involving the treatment of growing patients. Additionally, the analyzed studies varied in their comparison parameters for the control group, with some articles using other functional therapies and others using untreated patients. More prospective, randomized clinical studies are recommended to explore the efficacy of elastodontic appliances compared to untreated control groups and control groups treated with functional appliances, which are well established and widely supported by scientific evidence.

## 5. Conclusions

Treatment with elastodontic appliances shows significant improvements in various cephalometric and dentoalveolar parameters, particularly ANB, SNB, and OVJ, indicating mandibular advancement or at least a forward repositioning of the mandible. Included studies suggest that these devices can be effective in correcting skeletal and dental relationships. However, the variability in the results underscores the need for further research to confirm these findings.

Additionally, the advantages of EAs over traditional functional appliances are not entirely clear and warrant more detailed evaluation.

## Figures and Tables

**Table 1:** Search strategy for Scopus, Web of Science, Embase, and PubMed.

Database	Search Strategy	Number of Results
Scopus	(TITLE-ABS-KEY ((“children” OR “mixed dentition” OR “deciduous dentition” OR “primary dentition” OR “deciduous teeth” OR “primary teeth”)) AND TITLE-ABS-KEY ((“elastodontic” OR “myofunctional” OR “prefabricated functional appliance” OR “myobrace”)))	504
Web of Science	(“children” OR “mixed dentition” OR “deciduous dentition” OR “primary dentition” OR “deciduous teeth” OR “primary teeth”) (Topic) and (“elastodontic” OR “myofunctional” OR “prefabricated functional appliance” OR “myobrace”) (Topic)	446
Embase	(‘children’/exp OR ‘children’ OR ‘mixed dentition’/exp OR ‘mixed dentition’ OR ‘deciduous dentition’/exp OR ‘deciduous dentition’ OR ‘primary dentition’/exp OR ‘primary dentition’ OR ‘deciduous teeth’/exp OR ‘deciduous teeth’ OR ‘primary teeth’/exp OR ‘primary teeth’) AND (‘elastodontic’ OR ‘myofunctional’ OR ‘prefabricated functional appliance’ OR ‘myobrace’)	426
PubMed	(“children” OR “mixed dentition” OR “deciduous dentition” OR “primary dentition” OR “deciduous teeth” OR “primary teeth”) AND (“elastodontic” OR “myofunctional” OR “prefabricated functional appliance” OR “myobrace”)	286

**Table 3:** Sample and treatment characteristics.

Author (Year) [Reference]	Sample: M/F (Age)	Sample Size Calculation	Malocclusion	Intervention (Device and Wear Instructions)	Comparison	Compliance	Follow-Up
Chen LR et al. (2022) [23]	Test group: 13: 9M/4F (9.3 years) Control group: 13: 9M/4F (9.9 years)	No	Class II div 1	PMA 2 h/daytime + all night Breathing exercise Lip exercise Tongue exercise	Untreated patients	NA	1 year
Ciavarella D et al. (2021) [16]	Test group: 20: 9M/11F (9.4 ± 0.3 years) Control group: 20: 7M/13F (9.7 ± 0.4 years)	No	Class II	PMA AMCOP 4 h/daytime + all night myofunctional exercises	Untreated patients	NA	2 years
Ciftci V et al. (2021) [26]	Test group: 18: 8M/10F (9.97 ± 1.36 years) Control group: NA	Yes	Class II div 1	Multi-P myofunctional appliance 4 h/daytime + all night	No	Yes	2.94 ± 0.70 years
Çoban Büyükbayraktar Z et al. (2023) [27]	Test group: 18: NR (12.14 ± 1.23 years) Control group: 18: NR (12.14 ± 1.23 years)	Yes	Class II	Myobrace N/R	Twin-block N/R	NA	6 months
Fichera G et al. (2021) [12]	Test group: 20: 8M/12F (8.4 ± 0.6years) Control group: 20: 9M/11F (8.1 ± 0.8 years)	No	Class II	AMCOP second class 1 h/daytime + all night	Untreated patients	NA	1 year
Galluccio G et al. (2021) [17]	Test group 3 (Oclusoguide): 24: 13M/11F (9.05 ± 0.39 years) Control group 1 (Frankel): 23: 14M/9F (10.3 ± 1.08 years) Control group 2 (twin-block): 18: 10M/8F (10.7 ± 1.05 years) Control group 3 (untreated): 20: 11M/9F (12.17 ± 1.7 years)	No	Class II div 1	PMA Occlus-o-Guide N/R	Control group 1: Fränkel-2 appliance N/R Control group 2: Twin-block N/R Control group 3: Untreated patients	NA	12 months
Inchingolo AD et al. (2022) [18]	Test group: 21: 10M/11F (8.22 ± 1.17 years) Control group: NA	No	Class II	AMCOP Integral AMCOP second class AMCOP Open 1 h/daytime + all night for 6–8 months, and then only at night	N/R	NA	16–18 months
Johnson JS et al. (2021) [29]	Test group 1 (myobrace) 10: N/R (10.40 ± 1.89 years) Control group 1 (twin-block): 10: N/R (10.850 ± 1.37 years) Control group 2 (untreated): 10: N/R (10.60 ± 1.77 years)	No	Class II div 1	Myobrace 1–2 h/daytime + 10–12 h/night	Control Group 1: Twin-block 24 h/day Control group 2: untreated patients	N/R	18–24 months
Lanteri V et al. (2022B) [19]	Test group: 36: 17M/19F (7.9 ± 0.7 years) Control group: 33: 15M/18F (7.7 ± 0.5 years)	Yes	Class I Class II	Customized eruption guide appliance 2 h/daytime + all night	Preformatted eruption guide appliance 2 h/daytime + all night	NA	1 year
Lo Giudice A et al. (2022) [20]	Test group: 19: 9M/10F (9.1 ± 0.7 years) Control group: 17: 7M/10F (8.8 ± 0.8 years)	Yes	Class II	AMCOP second class 1 h/daytime + all night Lip exercises	Untreated patients	N/R	1 year
Madian AM et al. (2023) [30]	Test group: 13 (9–12 years) Control group: 13 (9–12 years)	Yes	Class II	Myobrace A minimum of 1–2 h per day and overnight	Twin-block All times except for eating	Yes	6 months
Patano A et al. (2023) [21]	Test group: 33: 14M/19F (8.9 ± 1.6 years) Control: 35: 18M/17F (8.9 ± 0.4 years)	No	Class II	AMCOP bioactivators 1 h during the day and throughout the night for 6–8 months, and then only at night	Untreated patients	NA	3 years (including treatment)
Ronsivalle V et al. (2023) [22]	Test group: 10: 5M/5F (7.5 ± 0.9 years) Control: 10 (7M/3F) (6.9 ± 1 years)	Yes	Class III	Class III elastodontic mono-block appliance AMCOP Class III activator At night and for two hours during the day	Bi-maxillary plates with class III elastics At night and for two hours during the day	NA	1 year
Usumez S et al. (2004) [28]	Test group: 20: 10M/10F (9.6 ± 1.3 years) Control: 20: 10M/10F (10.2 ± 0.8 years)	No	Class II div 1	Preorthodontic trainer appliance (Myofunctional Research Co., Queensland, Australia) Every day for one hour and overnight	Untreated patients	NA	-
Yang X et al. (2022) [24]	Test group: 56: 30/26 (8.1 ± 1.1 years) Control: 53: 25/28 (8.2 ± 1.0 years)	No	Class I or II	Preformed appliances (MRC Myofunctional Research Co. Queensland, Australia) At night (≥ 8 h) during sleep and continuously for 2 h during the day	Conventional early orthodontic appliances (arch expansion devices along with “2 × 4” local fixed appliances)	NA	2 years
Zhang X et al. (2021) [25]	Test group: 14 (9.2 years) Control: 14 (10 years)	No	Class II div 1	T4K At least 14 h per day (overnight use and at least two hours during the day)	Hyrax appliance	NA	1 year

AMCOP, Armonizzatori Multifunzionali Cranio-Occluso Posturali; F, female; h, hours; M, male; NA, not applicable; NR, not reported; PMA, prefabricated myofunctional appliance; div, division.

**Table 4:** Intragroup differences (T1 − T0) and intergroup differences (T1 − T0) in the cephalometric values analyzed.

Author (Year) [Reference]	Intragroup Meas. Difference (T1 − T0)/*p* Value		Intergroup Meas. Difference (Elastomeric Appliance − Control) *p* Value
	Elastomeric Appliance Group	Control Group	
Chen LR et al. (2022) [23]	SNA: 0.80 (1.71)/0.106 SNB: 1.87 (1.89)/**0.004** ANB: −1.15 (1.15)/**0.011** Ar-B: 3.61 (2.33)/**0.002** OVB: NR OVJ: NR FMA: −0.25 (2.31)/0.664 SN-Go-Gn: −0.85 (3.11)/0.305	SNA: 0.91 (2.20)/0.150 SNB: 0.95 (1.10)/**0.013** ANB: −0.12 (2.11)/0.325 Ar-B: 3.68 (4.07)/**0.003** OVB: NR OVJ: NR FMA: 0.82 (3.40)/0.477 SN-Go-Gn: −0.60 (1.94)/0.154	SNA: 0.917 SNB: 0.143 ANB: 0.164 Ar-B: 0.606 OVB: NR OVJ: NR FMA: 0.680 SN-Go-Gn: 0.408
Ciavarella D et al. (2021) [16]	SNA: NR SNB: NR ANB: NR Co-Gn: 9.3**/≤0.05** OVB: NR OVJ: NR FMA: NR SN-Go-Me: −1/NS	SNA: NR SNB: NR ANB: NR Co-Gn: −0.5/NS OVB: NR OVJ: NR FMA: NR SN-Go-Me: −0.85/≤0.01	SNA: NR SNB: NR ANB: NR Co-Gn: **0.0173** OVB: NR OVJ: NR FMA: NR SN-Go-Me: 0.3378
Ciftci V et al. (2021) [26]	SNA: −0.96/0.346 SNB: 1.3/0.236 ANB: −1.6/**0.001** Go-Gn: 3.66/**0.030** Co-Gn: 1.51/0.833 OVB: 1.12/0.261 OVJ: −4.58/**0.000** FMA: −1.9/0.082 SN-Go-Gn: −0.91/0.451	NR	NR
Çoban Büyükbayraktar Z et al. (2023) [27]	SNA: 0.20 (1.04)/0.415 SNB: 0.61 (0.80)/**0.004** ANB: −0.38 (1.19)/0.178 Go-Pg: 1.61 (5.14)/0.188 Co-Gn: 2.31 (8.4)/0.248 OVB: −0.10 (2.27)/0.407 OVJ: −2.92 (3.47)/**0.001** FMA: 0.66 (3.07)/0.360 SN-Go-Gn: 0.66 (1.84)1/0.429	SNA: −0.44 (0.97)/**0.001** SNB: 1.31 (1.42)/**0.001** ANB: −1.75 (1.08)/**0.001** Go-Pg: 2.68 (3.70)/**0.01** Co-Gn: 4.77 (4.60)/**0.003** OVB: −0.21 (1.94)/0.255 OVJ: −2.99 (1.93)/0.216 FMA: 1.65 (2.95)/**0.001** SN-Go-Gn: 0.96 (1.44)/**0.001**	SNA: 0.554 SNB: 0.52 ANB: 0.608 Go-Pg: 0.861 Co-Gn: 0.310 OVB: 0.502 OVJ: 0.331 FMA: 0.703 SN-Go-Gn: 0.409
Fichera G et al. (2021) [12]	SNA: 0.66/NS SNB: 2.7/**<0.05** ANB: −1.94/**<0.05** OVB: −2.6/**<0.05** OVJ: −2.6/**<0.05** FMA: NR SN-Go-Gn: NR	SNA: NR SNB: NR ANB: NR OVJ: 0.3/NS OVB: 0.7/**<0.05** FMA: NR SN-Go-Gn: NR	SNA: NR SNB: NR ANB: NR OVJ: NR OVB: NR FMA: NR SN-Go-Gn: NR
Galluccio G et al. (2021) [17]	SNA: −0.58 (1.89)/0.178 SNB: 1.42 (2.08)/**0.002** ANB: −1.96 (1.12)/**<0.001** Ar-Pg: 6.13 (3.11)/**<0.001** OVB: −1.04 (1.27)/**0.002** OVJ: −3.13 (1.85)/**<0.001** FMA: NR SN-Go-Gn: −0.38 (2.81)/0.531	Control Group 1 (FR-2): SNA: −0.09 (0.996)/0.68 SNB: 2.13 (0.97)/**<0.001** ANB: −2.17 (0.78)/**<0.001** Ar-Pg: 6.74 (2.68)/**<0.001** OVB: −1.65 (1.87)/**0.001** OVJ: −3.17 (1.92)/**<0.001** FMA: NR SN-Go-Gn: −0.57 (2.86)/0.28 Control Group 2 (TB): SNA: −0.17 (0.86)/0.298 SNB: 2.28 (1.07)/**<0.001** ANB: −2.33 (1.03)/**<0.001** Art-Pg: 6.78 (1.55)/**<0.001** OVB: −2.22 (1.06)/**<0.001** OVJ: −4.28 (0.89)/**<0.001** FMA: NR SN-Go-Gn: 0.33 (3.03)/0.428 Control Group 3 (Untreated): SNA: 0.41 (0.4) SNB: 0.7 (0.5) ANB: −0.2 (0.1) Art-Pg: 4.1 (3.2) OVB: 0.3 (0.6) OVJ: 0.1 (0.1) FMA: NR SN-Go-Gn: −0.7 (0.6)	Occlus-o-Guide vs. Control group SNA **0.017** SNB: 0.105 ANB: **<0.001** Ar-Pg: **0.004** OVB: **<0.001** OVJ: **<0.001** FMA: NR SN-Go-Gn: 0.576 Occlus-o-Guide vs. TB SNA: NR SNB: NR ANB: NR Art-Pg: NR OVB: **0.041** OVJ: **0.02** FMA: NR SN-Go-Gn: NR
Inchingolo AD et al. (2022) [18]	SNA: −0.04/0.9484 SNB: 2.17/**0.0015** ANB: −2.28/**0.0001** OVB: 2.12/0.1245 OVJ: −2.64/**0.0002** FMA: NR SN-Go-Gn: −2.87/**0.0014**	NR	NR
Johnson JS et al. (2021) [29]	SNA: −0.09 (0.62)/0.661 SNB: 1.35 (0.97)/**0.002** ANB: −1.14 (1.33)/**0.024** Go-Me: 1.75 (0.97)/**0.000** Ar-Gn: 1.55 (0.76)/**0.000** OVB: −0.08 (1.17)/0.834 OVJ: −3.55 (2.59)/**0.002** FMA: NR SN-Go-Gn: −0.70 (0.88)/0.034	Control group 1 (TB): SNA: −0.30 (0.48)/0.081 SNB: 2.00 (1.33)/**<0.001** ANB: −2.20 (1.22)/**0.000** Go-Me: 3.90 (2.95)/**0.002** Ar-Gn: 4.60 (4.56)/**0.011** OVB: −1.25 (1.03)/**0.004** OVJ: −5.10 (3.07)/**0.001** FMA: NR SN-Go-Gn: 0.18 (0.49)/0.281 Control group 2 (Untreated): SNA: −0.01 (0.63)/0.961 SNB: 0.30 (0.35)/**<0.001** ANB: −0.21 (0.72)/0.386 Go-Me: 0.50 (0.70)/0.052 Ar-Gn: 0.20 (0.34)/0.104 OVB: 0.34 (0.47)/**0.049** OVJ: 0.19 (1.15)/0.616 FMA: NR SN-Go-Gn: −0.34 (0.73)/0.178	Myobrace vs. TB: SNA: 0.489 SNB: 0.176 ANB: 0.053 Go-Me: 0.107 Ar-Gn: **0.037** OVB: **0.039** OVJ: 0.148 FMA: NR SN-Go-Gn: **0.026** Myobrace vs. control: SNA: 0.875 SNB: **0.001** ANB: 0.101 Go-Me: **0.005** Ar-Gn: **<0.001** OVB: 0.314 OVJ: **<0.001** FMA: NR SN-Go-Gn: 0.421
Lanteri V et al. (2022B) [19]	SNA: 0.79/0.14 SNB: 2.42/**<0.05** ANB: −1.43/**<0.05** OVB: −1.86/**<0.01** OVJ: −2.36/**<0.01** FMA: NR SN-Go-Gn: 2.23/**<0.05**	SNA: 0.83/0.12 SNB: 1.75/**<0.05** ANB: 0.97/**<0.05** OVB: −1.24/**<0.05** OVJ: −2.22/**<0.01** FMA: NR SN-Go-Gn: 0.98/0.083	SNA: 0.33 SNB: 0.09 ANB: 0.17 OVB: **<0.05** OVJ: 0.08 FMA: NR SN-Go-Gn: <0.05
Lo Giudice A et al. (2022) [20]	SNA: 0.4/NS SNB: 2.4/**<0.05** ANB: −2.1/**<0.05** OVB: −2.2/**<0.05** OVJ: −2.8/**<0.05** FMA: NR SN-Go-Gn: NR	SNA: NR SNB: NR ANB: NR OVB: 0.5/NS OVJ: 0.6/NS FMA: NR SN-Go-Gn: NR	Matching Percentage (**<0.05**)
Madian AM et al. (2023) [30]	SNA: 0.64 (1.08)/0.06 SNB: 2.82 (3.32)/**0.01** ANB: −2.42 (2.70)/**0.007** OVB: NR OVJ: NR FMA: 0.80 (4.00)/0.49 SN-Go-Gn: NR	SNA: −0.03 (0.47)/0.82 SNB: 3.79 (3.06)/**0.001** ANB: −3.06 (1.14)/**<0.001** OVB: NR OVJ: NR FMA: −2.69 (5.96)/0.13 SN-Go-Gn: NR	SNA: 0.06 SNB: 0.45 ANB: 0.43 OVB: NR OVJ: NR FMA: 0.09 SN-Go-Gn: NR
Patano A et al. (2023) [21]	SNA: −1/**0.0053** SNB: 1.3/**<0.0001** ANB: −2.2/**<0.0001** Co-Me: 8.5/**<0.0001** OVB: 0.3/0.5079 OVJ: −2.1/**<0.0001** FMA: −0.8/0.0773 SN-Go-Gn: −0.2/0.5227	SNA: 0.4/0.2515 SNB: 0.6/0.0617 ANB: −0.2/0.5204 Co-Me: 5.4/**<0.0001** OVB: 1.4/**0.0001** OVJ: 0.2/0.2132 FMA: −0.9/**0.0221** SN-Go-Gn: −0.4/0.2062	SNA: 0.008 SNB: 0.102 ANB: **<0.001** Co-Me: 0.102 OVB: **0.01** OVJ: **<0.001** FMA: 0.915 SN-Go-Gn: 0.8
Ronsivalle V et al. (2023) [22]	SNA: 0.8/0.071 SNB: −1.8/**<0.05** ANB: 2.6/**<0.05** OVB: NR OVJ: 3.1/**<0.05** FMA: NR SN-Go-Gn: NR	SNA: 1/0.084 SNB: −2.1/**<0.05** ANB: 3.1/**<0.05** OVB: NR OVJ: 2.9/**<0.05** FMA: NR SN-Go-Gn: NR	SNA: 0.168 SNB: 0.211 ANB: 0.114 OVB: NR OVJ: 0.163 FMA: NR SN-Go-Gn: NR
Usumez S et al. (2004) [28]	SNA: 0.13 (1.02)/NS SNB: 1.31 (1.35)/**0.001** ANB: −1.19 (1.18)/**0.001** Co-Gn: 2.88 (4.53)/**0.023** OVB: −0.22 (1.76)/NS OVJ: −3.75 (1.60)/**0.000** FMA: NR SN-Go-Gn: −1.50 (1.76)/0.003	SNA: −0.11 (1.70)/NS SNB: 0.41 (1.64)/NS ANB: −0.50 (1.24)/NS Co-Gn: 1.47 (2.60)/**0.021** OVB: 0.06 (0.39)/NS OVJ: −0.13 (0.78)/NS FMA: NR SN-Go-Gn: −0.34 (1.90)/NS	SNA: NS SNB: NS ANB: NS Co-Gn: NS OVB: NS OVJ: **0.000** FMA: NR SN-Go-Gn: NS
Yang X et al. (2022) [24]	SNA: 0.32 (1.98)/0.23 SNB: 1.06 (1.58)/**0.00** ANB: NR OVB: NR OVJ: NR FMA: 0.18 (2.12)/0.53 SN-Go-Gn: NR	SNA: −0.02 (1.82)/0.94 SNB: 0.43 (1.55)/**0.05** ANB: NR OVB: NR OVJ: NR FMA: −0.02 (2.64)/0.96 SN-Go-Gn: NR	SNA: 0.35 SNB: **0.04** ANB: NR OVB: NR OVJ: NR FMA: 0.67 SN-Go-Gn: NR
Zhang X et al. (2021) [25]	SNA: 1.39 (1.11)/**0.022** SNB: 2.48 (1.27)/**0.003** ANB: −1.06 (1.10)/0.055 Go-Me: 3.50 (2.07)/**0.006** Pg/OLP: 3.41 (2.04)/**0.006** OVB: NR OVJ: 0.81 (3.54)/0.594 FMA: NR SN-Go-Gn: NR	SNA: 0.05 (1.39)/0.906 SNB: 0.39 (1.50)/0.39 ANB: −0.34 (1.17)/0.337 Go-Me: 1.00 (2.09)/0.124 Pg/OLP: 2.33 (3.19)/**0.039** OVB: NR OVJ: −0.92 (2.28)/0.186 FMA: NR SN-Go-Gn: NR	SNA: **0.013** SNB: **0.001** ANB: 0.123 Go-Me: **0.006** Pg/OLP: 0.239 OVB: NR OVJ: 0.161 FMA: NR SN-Go-Gn: NR

ANB, skeletal class, Point A–Nasion–Point B angle; Ar-B, mandibular length, Articulare–B point; Ar-Gn, mandibular length, Articulare–Gnathion; Ar-Pg, mandibular length, Articulare–Pogonion; Co-Gn, mandibular length, Condylion–Gnathion; Co-Me, mandibular length, Condylion–Menton; FMA, Frankfort plane and mandibular plane (Go-Gn); Go-Me, corpus length, Gonion–Menton segment; Go-Pg, mandibular length, Gonion–Pogonion; NR, not reported; NS, not significant; OVB, overbite; OVJ, overjet; Pg/OLP, sagittal mandibular position, Pogonion–perpendicular occlusal plane passing through Sella point; SNA, maxillary position, Sella–Nasion–Point A angle; SNB, mandibular position, Sella–Nasion–Point B angle; SN-Go-Gn, direction of mandibular growth relative to the cranial base, Sella–Nasion plane and Gonion–Gnathion plane; T0, pretreatment; T1, postreatment.

## Data Availability

The raw data supporting the conclusions of this article will be made available by the authors upon request.
